# Gut Microbiota Abrogates Anti-α-Gal IgA Response in Lungs and Protects against Experimental *Aspergillus* Infection in Poultry

**DOI:** 10.3390/vaccines8020285

**Published:** 2020-06-07

**Authors:** Lourdes Mateos-Hernández, Veronica Risco-Castillo, Edgar Torres-Maravilla, Luis G. Bermúdez-Humarán, Pilar Alberdi, Adnan Hodžić, Angelica Hernández-Jarguin, Sabine Rakotobe, Clemence Galon, Elodie Devillers, Jose de la Fuente, Jacques Guillot, Alejandro Cabezas-Cruz

**Affiliations:** 1 UMR BIPAR, INRAE, ANSES, Ecole Nationale Vétérinaire d’Alfort, Université Paris-Est, 14 rue Pierre et Marie Curie, 94706 Maisons-Alfort, France; lourdes.mateos@vet-alfort.fr (L.M.-H.); sabine.rakotobe@anses.fr (S.R.); clemence.galon@anses.fr (C.G.); elodie.devillers@anses.fr (E.D.); 2 EA 7380 Dynamyc, UPEC, USC, ANSES, Ecole Nationale Vétérinaire d’Alfort, Université Paris-Est, 94700 Maisons-Alfort, France; veronica.risco-castillo@vet-alfort.fr (V.R.-C.); jacques.guillot@vet-alfort.fr (J.G.); 3 Micalis Institute, AgroParisTech, INRAE, Université Paris-Saclay, 78350 Jouy-en-Josas, France; edgar.torres-maravilla@inrae.fr (E.T.-M.); luis.bermudez@inrae.fr (L.G.B.-H.); 4 SaBio, Instituto de Investigación en Recursos Cinegéticos (IREC-CSIC-UCLM-JCCM), Ronda de Toledo s/n, 13005 Ciudad Real, Spain; maria.alberdi@uclm.es (P.A.); AngelicaM.Hernandez@uclm.es (A.H.-J.); jose_delafuente@yahoo.com (J.d.l.F.); 5 Institute of Parasitology, Department of Pathobiology, University of Veterinary Medicine Vienna, 1210 Vienna, Austria; Adnan.Hodzic@vetmeduni.ac.at; 6 Facultad de Medicina Veterinaria y Zootecnia, Universidad Autónoma de Tamaulipas, Tamaulipas 87000, Mexico; 7 Department of Veterinary Pathobiology, Center for Veterinary Health Sciences, Oklahoma State University, Stillwater, OK 74078, USA

**Keywords:** alpha-Gal, microbiota, aspergillosis, *Aspergillus fumigatus*, granulomas, cross-protective immunity

## Abstract

Naturally occurring human antibodies (Abs) of the isotypes IgM and IgG and reactive to the galactose-α-1,3-galactose (α-Gal) epitope are associated with protection against infectious diseases, caused by pathogens expressing the glycan. Gut microbiota bacteria expressing α-Gal regulate the immune response to this glycan in animals lacking endogenous α-Gal. Here, we asked whether the production of anti-α-Gal Abs in response to microbiota stimulation in birds, confers protection against infection by *Aspergillus fumigatus*, a major fungal pathogen that expresses α-Gal in its surface. We demonstrated that the oral administration of *Escherichia coli* O86:B7 strain, a bacterium with high α-Gal content, reduces the occurrence of granulomas in lungs and protects turkeys from developing acute aspergillosis. Surprisingly, the protective effect of *E. coli* O86:B7 was not associated with an increase in circulating anti-α-Gal IgY levels, but with a striking reduction of anti-α-Gal IgA in the lungs of infected turkeys. Subcutaneous immunization against α-Gal did not induce a significant reduction of lung anti-α-Gal IgA and failed to protect against an infectious challenge with *A. fumigatus*. Oral administration of *E. coli* O86:B7 was not associated with the upregulation of lung cytokines upon *A. fumigatus* infection. We concluded that the oral administration of bacteria expressing high levels of α-Gal decreases the levels of lung anti-α-Gal IgA, which are mediators of inflammation and lung damage during acute aspergillosis.

## 1. Introduction

Galactose-α-1,3-galactose (α-Gal) is an oligosaccharide abundantly expressed on the glycoproteins and glycolipids of non-primate mammals, prosimians, and New World monkeys and is synthesized by the enzyme α-1,3-galactosyltransferase, encoded by the gene *ggta1* [1]. Non-mammalian vertebrates including fish, amphibians, reptiles and birds do not express the α-Gal epitope [1,2,3]. Humans, apes and Old World monkeys do not synthetize α-Gal, presumably due to the functional inactivation of the gene *ggta1* in the common ancestor of these animals [3], which resulted in the capacity to produce large amounts of antibodies (Abs) against the glycan epitope [4]. Bacteria from human gut microbiota also express α-Gal on their surface and a continuous antigenic stimulation produce serum accumulation of natural anti-α-Gal immunoglobulins G (IgG) and IgM [3,5]. Gut colonization by the bacterium *Escherichia coli* O86:B7 [6], which expresses high levels of α-Gal [7,8], elicits the production of Abs with reactivity to α-Gal and α-Gal-related B blood group glycan in α-Gal-deficient mice [9], non-human primates [10], chickens [11] and humans [12].

Based on the protective role of anti-α-Gal Abs, it was suggested that the inactivation of *ggta1* was due to strong selective pressure exerted on primate ancestors by an infectious agent, expressing α-Gal [3]. In support to this hypothesis, gut colonization by *E. coli* O86:B7 elicits anti-α-Gal IgM that protected α-Gal-deficient mouse against malaria transmission by *Anopheles* mosquitoes [8]. High levels of anti-α-Gal IgG and IgM in humans were associated with protection to *Plasmodium* sp., a pathogen expressing the antigen α-Gal, in malaria endemic regions [7,8]. However, the α-Gal immunity evolved as a trade-off between the protection to pathogens expressing α-Gal, which is mediated by anti-α-Gal IgG and IgM; and allergies, mediated by anti-α-Gal IgE [13]. Anti-α-Gal IgE production is associated with the onset of the α-Gal syndrome (AGS), a type of allergy, triggered by tick bites and characterized by delayed hypersensitivity to consumed red meat products in humans [14,15,16,17,18,19]. Ticks synthetize α-Gal [20], and several tick proteins in the saliva and cement have this glycan modification [17,20,21,22,23].

Aspergillosis, produced by the saprophytic opportunist fungus *Aspergillus fumigatus* with α-Gal on its surface [24], is one of the most prevalent airborne fungal infections affecting humans and animals worldwide [25,26,27]. *Aspergillus fumigatus* can cause a life-threatening disease in immunosuppressed and vulnerable individuals. Clinical presentation of aspergillosis varies according to the infectious load and the immunocompetence of the host. In humans, acute aspergillosis emerges as one of the first medical concerns in immunocompromised patients, especially those submitted to bone marrow or solid-organ transplantation or patients with cancer or HIV [25,27]. Despite the infective stage of *A. fumigatus*, the conidia, expresses high levels of α-Gal on its outer wall and the epitope is exposed to the host immune system; no difference in the levels of circulating anti-α-Gal IgG, IgM and IgE was found in patients with invasive aspergillosis and healthy control individuals [24]. In animals, vulnerability to *Aspergillus* infection varies among host species, with birds exhibiting the highest susceptibility. Among galliform species, infected turkey poults have high morbidity and mortality rates [26]. Clinical signs are usually unexpected and particularly severe, and mortality remains high even after antifungal treatment [28]. Lung damage is commonly found in several forms of aspergillosis in birds [29,30] and humans [31]. The gut-lung microbiota axis may influence the pathogenesis of aspergillosis [32]. However, the mechanisms by which microbiota drives the pathogenesis of aspergillosis are poorly understood [32]. Particularly, the capacity of gut microbiota to elicit anti-α-Gal IgA in the lungs, and the role of these Abs as mediators of inflammation and immunity in human and non-mammalian vertebrates have not been explored in the context of aspergillosis.

To address this gap, experimentally *Aspergillus*-infected turkeys *Meleagris gallopavo* and chickens *Gallus gallus domesticus* [30,33,34] were used in this study, to explore the role of gut microbiota and anti-α-Gal immunity for the control of aspergillosis. The results of this study showed that gut microbiota enriched with bacteria expressing high levels of α-Gal protects turkeys against clinical aspergillosis and the formation of lung granulomas, by reducing lung anti-α-Gal IgA to residual levels.

## 2. Materials and Methods

### 2.1. Ethics Statement

All procedures in this work were performed according to the principles established by the French and International Guiding Principles for Biomedical Research Involving Animals (2012). The regional ethics committee for animal experimentation at the Veterinary College of Alfort approved this research (Anses/EnvA/UPEC, approval No. 10/03/15-11).

### 2.2. Environmental Contamination Assessment

The absence of fungal contamination was assessed in the environment, litter and feedstuff using malt-chloramphenicol agar plates (AEMTEK, Fremont, CA, USA). Fungal contamination of the environment was assessed before starting and during the experiment by sampling of conidia by sedimentation on two opened agar plates placed in different points of the room for 30 min. Litter and feedstuff (1 g each) were mixed in 45 mL of phosphate-buffered saline (PBS, 10 mM NaH_2_PO_4_, 2.68 mM KCl, 140 mM NaCl, pH 7.2, Thermo Scientific, Waltham, MA, USA) containing 0.01% (vol/vol) Tween 20 (PBST) and 100 µL of the mix were seeded in agar plates. All plates were incubated at 37 °C during at least 48 h.

### 2.3. Animals and Housing Conditions

One-day-old female turkeys Hybrid Diamond White Medium strain (Grimaud Frères Sélection, La Corbière, France) and chickens Lohmann Brown strain (Lohmann France, Le Grand Moulin, France) were purchased with an average weight of 65–70 g and 40–50 g, respectively. The animals were housed in cages (Ducatillon, Cysoing, France) under specific-pathogen-free (SPF) conditions in the biosafety level 3 sector of the animal facility of the Veterinary College of Alfort (CRBM-EnvA, Maisons-Alfort, France). Fresh commercial turkey feed ‘dindonneau pintadeau uni S25’ (Axereal, Olivet, France, ref. 1930920) and chicken starter feed ‘gold 1&2 crumble 5 kg’ (Versele-Laga, Deinze, Belgium, ref. 283.0250) and fresh water were provided *ad libitum*. The photoperiod cycles (14 h per day) and room temperature (25 °C) were controlled. Additional heat was provided by two infrared lamps located close to the animals. At no point were the animals used in this study placed under antibiotic treatment.

### 2.4. Aspergillus fumigatus Strain and Inoculum Preparation

The highly germinative *A. fumigatus* CBS 144.89 (CEA10) clinical strain was used for all experiments [35]. All mycological cultures were performed on Sabouraud dextrose agar (SDA), supplemented with chloramphenicol (5 mg/L) and incubated at 37 °C for 10 days. Sub-cultures were performed twice a week. To prepare the inoculum, *A. fumigatus* colonies were grown for 2–3 days at 37 °C. Conidia were subsequently harvested by resuspension in PBST, filtered in a 70 µm diameter nylon cell strainer (ClearLine Dominique Dutscher, Brumath, France), washed by centrifugation at 3500× *g* for 10 min, resuspended in PBST and then counted using a Malassez counting chamber. The inoculum of *A. fumigatus* contained 4 × 10^7^ conidia resuspended in 200 µL of PBST [30]. All reagents used for inoculum preparation were apyrogenic.

### 2.5. Detection of α-Gal Glycan in Fungi

The presence of α-Gal in *A. fumigatus* (Ascomycota) was detected by immunofluorescence, flow cytometry and inhibition ELISA. The presence of α-Gal modification in proteins of other Ascomycota (i.e., *Aspergillus nidulans*, *Candida glabrata*, *Candida albicans*, *Microsporum canis*, *Penicillium* sp., *Scedosporium* sp., and *Trichophyton benhamiae*) and Zygomycota (i.e., *Mucor* sp. and *Rhizopus* sp.) fungi was assessed by inhibition ELISA.

For immunofluorescence, *A. fumigatus* conidia were cultured as described above and hyphae were separated from SDA media using PBS by gently scrapping. Conidia and hyphae were washed in PBS and then fixed and permeabilized with the Intracell fixation and permeabilization kit (Immunostep, Salamanca, Spain), following manufacturer recommendations. Fixed conidia and hyphae were incubated for 1 h at room temperature (RT) with 3% Human Serum Albumin (HSA, Sigma-Aldrich, St. Louis, MO, USA) in PBS. The monoclonal mouse anti-α-Gal antibody (mAb) M86 (Enzo Life Sciences, Farmingdale, NY, USA) diluted 1:50 in 3% HSA/PBS was used as primary Ab (incubation for 14 h at 4 °C) and the FITC-conjugated goat anti-mouse IgM (Abcam, Cambridge, UK) diluted 1:200 in 3% HSA/PBS as a secondary Ab (incubation for 1 h at RT). Hyphal mitochondria were stained with Mitotracker Red (Thermo Scientific, Waltham, MA, USA). Aliquots of fixed and stained conidia were used for immunofluorescence assays, mounted in glass slides using ProLong Antifade (Thermo Scientific, Waltham, MA, USA) with DAPI reagent (Molecular Probes, Eugene, OR, USA) and examined using a Zeiss LSM 800 laser scanning confocal microscope (Carl Zeiss, Oberkochen, Germany) with oil immersion objectives.

The detection of α-Gal by flow cytometry was performed as previously described [20,24]. Briefly, samples were analyzed on a FACSCalibur flow cytometer equipped with CellQuest Pro software (BD Bio-Sciences, Madrid, Spain). The cell population was gated according to forward-scatter and side-scatter parameters. The human promyelocytic leukemia HL60 cells, that do not express α-Gal, were included as a negative control. The mean and median fluorescence intensity of HL60 and conidia was recorded and compared.

Fungal proteins were extracted with six steel balls using the homogenizer Precellys^®^24 Dual (Bertin, Montigny-le-Bretonneux, France) at 6000 rpm for 30 s, followed by cool down in ice, 3 times in PBS-1% triton and quantified by Bicinchoninic Acid (BCA) Protein Assay Kit (ThermoFisher, Waltham, MA, USA) with Bovine Serum Albumin (BSA) as standard.

For inhibition ELISA, the inhibition of M86 binding to Galα1-3Gal linked to HSA (Galα1-3Gal-HSA, Dextra Laboratories, Reading, UK) was calculated after pre-incubation of M86 with fungal proteins. Briefly, 96-well ELISA plates (Nunc-ImmunoTM Plate, Roskilde, Denmark) were coated overnight at 4 °C with Galα1-3Gal-HSA (200 ng/well), diluted in carbonate/bicarbonate buffer (0.05 M, pH 9.6). The wells were washed three times with 150 µL of PBST and then blocked with 0.5% HSA/PBST for 1 h at RT. The mAb M86 diluted 1:200 was pre-incubated overnight at 4 °C and constant shaking of 300 rpm with two concentrations of fungal proteins (i.e., 0.5 µg/mL and 1.5 µg/mL). Pre-incubation with α-Gal-BSA and protein extract of *ggta1* knockout (KO) *Sus scrofa* (pig) were used as positive and negative controls, respectively. The protein-mAb M86 complexes were removed by centrifugation at 16,000× *g* for 30 min at 4 °C. The supernatant (containing free mAb M86) was then collected and added to the Galα1-3Gal-HSA-coated wells for 1 h at 37 °C. The plates were washed three times and horseradish peroxidase (HRP)-conjugated goat anti-mouse IgM Ab diluted 1:2000 was used as secondary Ab and incubated at RT for 1 h. The plates were washed three times and the reaction was developed by adding 100 µL ready-to-use tetramethylbenzidine-hydrogen peroxide (TMB) solution (Promega, Madison, WI, USA) at RT for 20 min in the dark, and then stopped with 50 µL of 0.5 M H_2_SO_4_. The optical densities (OD) were measured at 450 nm using an ELISA plate reader (Filter-Max F5, Molecular Devices, San Jose, CA, USA). All samples were tested in triplicate and the average value of three blanks (no Abs) was subtracted from the reads. The cut-off was determined as two times of a mean OD value of the blank controls. The percentage of M86-binding inhibition was calculated using the average OD of each sample as 100 − (100 × OD (M86 after pre-incubation with fungal proteins or α-gal-BSA or protein extract of *ggta1* KO pigs)/OD (M86 without pre-incubation)).

### 2.6. Bacteria Culture and Oral Administration of Bacteria

The bacterium *E. coli* O86:B7 (ATCC 12701) expresses high levels of α-Gal on its surface [7,8], which is not the case for *E. coli* BL21 (DE3, Invitrogen, Carlsbad, CA, USA) [7]. The *E. coli* strains were grown on 50 mL of Luria Broth (Sigma-Aldrich, St. Louis, MO, USA), incubated at 37 °C with vigorous shaking overnight, washed twice with PBS, centrifuged at 4000× *g* for 5 min at 4 °C and re-suspended at a concentration of ~1 × 10^10^ colony-forming units (CFU)/mL of PBS. For oral administration of bacteria, 7-day-old turkeys (*n* = 20) received *E. coli* strain O86:B7 (*n* = 10) or *E. coli* strain BL21 (*n* = 10) (~1 × 10^9^ CFU in 100 µL of PBS) via oral gavage at days 0, 1, 3, 7, 8, 9, 14, 15 and 16. All reagents used for bacterial preparation were apyrogenic.

### 2.7. Immunization

For immunization, 7-day-old turkeys (*n* = 20) and chickens (*n* = 10) were immunized subcutaneously with synthetic Galα1-3Gal conjugated to BSA (α-Gal-BSA, Dextra Laboratories, Reading, UK) (75 µg/bird), in 200 µL of the water-in-oil emulsion of 70% Montanide ISA adjuvant (SEPPIC, Castres, France), with a boost 2 weeks later (day 14). Control animals received a mock vaccine containing PBS and adjuvant.

### 2.8. Intratracheal Challenge with A. fumigatus

The intratracheal challenge was performed on day 27. Before fungal inoculation, birds were anesthetized by inhalation of 5% isoflurane (Aerrane, Baxter, Maurepas, France) in oxygen until unconsciousness. Inoculation of *A. fumigatus* was performed using a 1 mL syringe (Medallion, Merit Medical, The Netherlands), fitted with a stainless steel 19-gauge aerosolizer (Microsprayer IA-1B, Penn Century, Wyndmoor, PA, USA). The gauge was inserted through the oropharynx into the trachea under visual control. After challenge, birds were monitored twice a day on a daily basis. Respiratory signs of avian aspergillosis (i.e., open-mouthed breathing, gasping and hyperpnea) were recorded. Animals were sacrificed 4 days after challenge.

### 2.9. Euthanasia, Lung Lesions Score and Sample Collection

On day 31, four days after the infectious challenge, birds were anesthetized and euthanized by occipital sinus injection of 182.20 mg/kg of sodium pentobarbital (Dolethal, Vetoquinol, Lure, France). The respiratory tract was removed aseptically under a laminar flow cabinet and the presence and size of lesions in the right and left lungs were registered. Observed lung lesions varied among congestive, hemorrhagic or consolidated/indurated lesions. They were classified according to the following score: no lesions (minimum score, 0); small lesions between 1 cm^2^ and 2 cm^2^ (score 1); moderate-size lesions between 3 cm^2^ and 4 cm^2^ (score 2) and extensive lesions with more than 4 cm^2^ and covering almost all the area of the lungs (maximum score, 3). The statistical differences between groups were evaluated using one-way ANOVA with Dunnett’s multiple comparison test applied for individual comparisons (for the groups treated with *E. coli* O86:B7, *E. coli* BL21 and PBS) and the unpaired non-parametric Mann–Whitney U test (for the groups immunized with α-Gal-BSA and the mock vaccine) in the GraphPad 5 Prism program (GraphPad Software Inc., San Diego, CA, USA). Differences were considered significant when *p* < 0.05.

Blood samples were collected on days 0, 7, 14 and 31 on sterile tubes without anticoagulant. For serum separation, the blood samples were incubated for 20–30 min at RT, allowing for clotting, and then centrifuged at 1500× *g* for 20 min at RT. After necropsy, samples from the right and left lungs were aseptically collected and conserved according to the analysis to be performed: samples for DNA (for *A. fumigatus 28S* quantification by quantitative PCR (qPCR)), RNA (for cytokines mRNA quantification by qPCR) and protein (for anti-α-Gal IgA quantification) extraction were placed immediately in liquid nitrogen; samples for histopathology were immediately fixed in 10% Neutral Buffered Formalin (NBF) and the samples for CFU assay were conserved in PBST on ice until processing. Ceca samples were also collected (for RNA extraction and cytokines quantification by qPCR) and placed immediately in liquid nitrogen.

### 2.10. Indirect ELISA for Anti-α-Gal IgY and IgA Levels Determination

To evaluate the levels of specific Abs against Galα1-3Gal and Galα1-3Galβ1-4GlcNAc in turkey and chicken sera, 96-well ELISA plates (Thermo Scientific, Waltham, MA, USA) were coated with 100 μL/well of either Galα1-3Gal-HSA and Galα1-3Galβ1-4GlcNAc linked to HSA (0.5 µg/mL, Dextra Laboratories, Reading, UK) and incubated overnight at 4 °C. The antigens were diluted in carbonate/bicarbonate buffer (0.05 M, pH 9.6) and incubated overnight at 4 °C. Optimal antigen concentration and dilutions of sera and conjugate were defined using a titration assay. Wells were washed three times with 150 µL of PBST and then blocked by adding 100 μL of 1% HSA/PBST for 1 h at RT. After three washes, serum samples, diluted in 0.5% HSA/PBST (1:500), were added to the wells and incubated for 1 h at 37 °C. The plates were washed three times and HRP-conjugated Abs (goat anti-turkey IgY) (Mybiosource, San Diego, CA, USA) goat anti-chicken IgY (Sigma-Aldrich, St. Louis, MO, USA) or goat anti-chicken IgA (CliniScience, Nanterre, France) were added at 1:2000 dilution in 0.5% HSA/PBST (100 μL/well) and incubated for 1 h at RT. The plates were washed three times and the reaction was developed by adding 100 µL ready-to-use TMB solution (Promega, Madison, WI, USA) at RT for 20 min in the dark, and then stopped with 50 µL of 0.5 M H_2_SO_4_. The OD were measured at 450 nm using an ELISA plate reader (Filter-Max F5, Molecular Devices, San Jose, CA, USA). All samples were tested in triplicate and the average value of three blanks (no Abs) was subtracted from the reads. The cut-off was determined as two times a mean OD value of the blank controls.

Determination of anti-α-Gal IgA levels in the lungs was performed as above, but using total lung proteins (600 ng), extracted by the TRI Reagent kit (Thermo Scientific, Waltham, MA, USA), following manufacturer recommendations. The statistical differences between groups were evaluated using one-way ANOVA with Dunnett’s multiple comparison test applied for individual comparisons in the GraphPad 5 Prism program (GraphPad Software Inc., San Diego, CA, USA). Differences were considered significant when *p* < 0.05.

### 2.11. Enzymatic Removal of α-Gal to Test the Specificity of Turkey Anti-α-Gal Abs

To assess the specificity of anti-α-Gal Abs in turkeys, the Galα1-3Gal-HSA antigen (Dextra Laboratories, Reading, UK) was immobilized on an ELISA plate (50 ng/well), and treated or not with α-galactosidase from green coffee beans (Sigma-Aldrich, St. Louis, MO, USA), following the procedure described elsewhere [36]. Before the treatment, the enzyme was centrifuged at 10,000× *g* for 10 min at 4 °C, to remove the ammonium sulfate. The supernatant was discarded and 100 mM potassium phosphate buffer (pH 6.5) was added to the pellet, so the final concentration of the enzyme solution was 50 mU/100 µL. The plate was then incubated at 37 °C for 24 h in a humidified plastic chamber to avoid evaporation. After the incubation, wells were washed five times with 150 µL of PBST and the indirect ELISA was performed as described above. Sera samples from the turkeys treated with *E. coli* O86:B7 (*n* = 5), *E. coli* BL21 (*n* = 5) and PBS (*n* = 5) were randomly selected and used in the specificity assay. The statistical differences of sera reactivity against treated and non-treated antigen were evaluated using the Wilcoxon signed rank test in the GraphPad 5 Prism program (GraphPad Software Inc., San Diego, CA, USA). Differences were considered significant when *p* < 0.05.

### 2.12. Histopathology and Histopathological Scores

Lung samples collected for histopathology were immediately fixed in 10% NBF for 48 h, then dehydrated in successive baths of ethanol (from 70 to 100%) and embedded in paraffin blocks using Fully Automated Innovative Tissue Processor LOGOS One (Milestone, Sorisole, Italy). Thick sections of 4 µm were cut out of the paraffin specimens and placed in slides. The slides were automatically stained with hematoxylin-eosin-saffron (HES), using Leica ST5010-CV5030 Integrated Workstation (Leica, Nanterre, Germany). Periodic Acid-Schiff (PAS) staining was performed using the PAS kit (Sigma-Aldrich, St. Louis, MO, USA), following the manufacturer’s instructions. A blind reading of five fields (100×) per slide of the right and left lungs of each turkey was conducted to visualize microscopic lesions associated with inflammation and granulomas (using slides stained with HES) and the presence of *Aspergillus*-like hyphae (using slides stained with PAS). Microscopic observations in each field were recorded and scored as follows for HES: absence of leukocyte infiltrate and peribronchial regions visible (minimum score, 0); leukocyte infiltrate surrounding peribronchial regions without lumen stenosis (score 1); intense leukocyte infiltrate and lumen of peribronchial regions is not visible (score 2); and the presence of granulomas (maximum score, 3)

Fungal presence by PAS was scored as follows: absence of fungal elements (minimum score, 0); presence of isolated germtube/hyphae (score 1), presence of several branching hyphae (mycelium) inside granulomas between 20 and 25 µm^2^ (score 2) and presence of mycelium inside granulomas larger than 25 µm^2^ (maximum score, 3). The scores of the five-field readings per slide and per staining were used to calculate the HES and PAS scores per lung and per animal. The statistical differences between groups were evaluated using one-way ANOVA with Dunnett’s multiple comparison test applied for individual comparisons (for the groups treated with *E. coli* O86:B7, *E. coli* BL21 and PBS) and the unpaired non-parametric Mann–Whitney U test (for the groups immunized with α-Gal-BSA and the mock vaccine) in the GraphPad 5 Prism program (GraphPad Software Inc., San Diego, CA, USA). Differences were considered significant when *p* < 0.05.

### 2.13. Quantification of A. fumigatus by CFU and qPCR Assays

For CFU counting, 100 mg of right lungs were individually ground in 5 mL of PBST using the Bio-Gen PRO200 Tissue Homogenizer (PRO Scientific, Oxford, CT, USA). An aliquot of the lung homogenate (100 µL) was immediately spread on SDA plates and incubated at 37 °C for 24 to 48 h, after which *A. fumigatus* colonies counting was performed. For qPCR, genomic DNA was extracted from 25 mg of the right lungs. The lung samples were individually crushed in 180 µL of Lysis Buffer (QIAamp DNA Mini Kit, Qiagen, Courtaboeuf, France) with glass beads using the Tissue Homogenizer 125 Precellys 24 (Bertin Technologies, Montigny-le-Bretonneux, France) at 6000 rpm for 30 s. The homogenization procedure was repeated three times with a 30 s cooling period in ice in between cycles. DNA extraction was completed using the QIAamp DNA Mini Kit (Qiagen, Courtaboeuf, France), according to the manufacturer’s instructions.

qPCR was performed with 100 ng of genomic DNA targeting *A. fumigatus 28S* gene with SYBR Green LightCycler 480 Master mix (Roche, Meylan, France). The selection of *A. fumigatus 28S* gene for fungal quantification was based on previously published research [30]. All assays were run under the same conditions as follow: 50 °C for 2 min, 95 °C for 10 min, and 45 cycles of 15 s at 95 °C and 1 min at 60 °C. The CT values were recorded, and the relative levels of fungal DNA were normalized against turkey β actin (*actb*) and glyceraldehyde-3-phosphate dehydrogenase (*gapdh*) and chicken *gapdh* as host genes. Relative quantification was achieved using the 2^−ΔΔ*C*t^ ratio method [37]. Primers were designed using Primer-BLAST online software [38] and are presented in Table 1. The statistical differences between groups were evaluated using one-way ANOVA with Dunnett’s multiple comparison test applied for individual comparisons (for the groups treated with *E. coli* O86:B7, *E. coli* BL21 and PBS) and the unpaired non-parametric Mann–Whitney U test (for the groups immunized with α-Gal-BSA and the mock vaccine) in the GraphPad 5 Prism program (GraphPad Software Inc., San Diego, CA, USA). Differences were considered significant when *p* < 0.05.

### 2.14. RNA Extraction and Quantification of Cytokines mRNA Levels by qPCR

Total RNA was extracted from 100 mg of right lung and ceca samples. Tissue samples were individually crushed in 1 mL of TRI Reagent (Thermo Scientific, Waltham, MA, USA) with glass beads using the Tissue Homogenizer 125 Precellys 24 (Bertin Technologies, Montigny-le-Bretonneux, France) at 6000 rpm for 30 s. The homogenization procedure was repeated three times. Complementary DNAs (cDNA) were obtained by reverse transcription of total RNA (1 µg) using random primers and the SuperScript VILO cDNA Synthesis Kit (Thermo Scientific, Waltham, MA, USA). Equal amounts of cDNA per sample (10 ng) were used in triplicate assays for qPCR amplification using the SYBR Green LightCycler 480 Master mix (Roche, Meylan, France) with 0.3 μM of each primer for the genes *IL2*, *IFNγ*, *MyD88*, *IL6* and *IL10* (Table 1). The LightCycler 480 System Thermocycler (Roche, Basilea, Suiza) was used. The qPCR assays were run under the following conditions: 50 °C for 2 min, 95 °C for 10 min, then 45 cycles of 15 s at 95 °C and 1 min at 60 °C. The CT values were recorded and the 2^−ΔΔ*C*t^ method [37] was used to calculate the relative gene expression values with turkey *actb* and *gapdh* and chicken *gapdh* as the endogenous control genes. Statistical differences between groups for each gene were evaluated using one-way ANOVA with Dunnett’s multiple comparison test applied for individual comparisons in the GraphPad 5 Prism program (GraphPad Software Inc., San Diego, CA, USA). Differences were considered significant when *p* < 0.05.

## 3. Results

### 3.1. A. fumigatus Contains the Carbohydrate α-Gal

**Figure 1:**
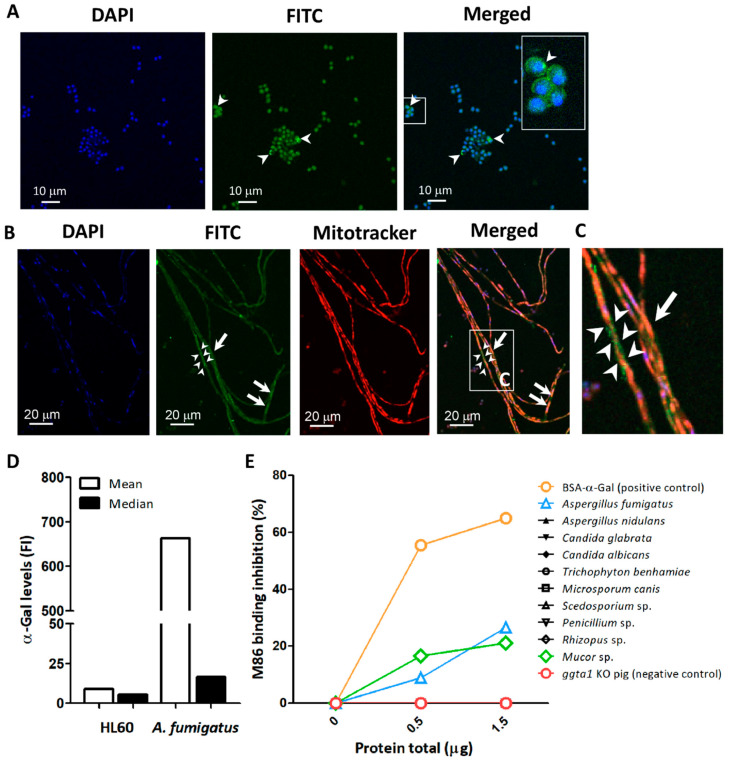
Detection of α-Gal in *A. fumigatus*. The α-Gal-specific mAb M86 (primary Ab) was used to detect the production of α-Gal in *A. fumigatus* conidia (arrow heads) (**A**) and hyphae (**B**) by immunofluorescence. The mAb M86 was reactive to granular structures surrounding the hyphae (arrow heads) and the cytoplasm of hyphae cells (arrows) (**B**,**C**). Goat anti-mouse IgM-FITC was used as secondary Ab for detection of α-Gal (green). Cell nuclei and mitochondria were stained with DAPI (blue) (**A**,**B**) and Mitotracker (red) (**B**), respectively. α-Gal expression in conidia surface was measured by flow cytometry using M86 and Goat anti-mouse IgM-FITC. Mean and median fluorescence intensity (FI) values are presented. HL60 cells were used as a negative control (**D**). Presence of α-Gal glycan in protein extract of Ascomycota (i.e., *A. fumigatus*, *A. nidulans*, *C. glabrata*, *C. albicans*, *M. canis*, *Penicillium* sp., *Scedosporium* sp., and *T. benhamiae*) and Zygomycota (i.e., *Mucor* sp. and *Rhizopus* sp.) fungi was measured by inhibition ELISA assay (**E**).

Immunofluorescence labeling using the anti-α-Gal mAb M86 [39] confirmed the presence of α-Gal glycan on the surface and cytoplasm of *A. fumigatus* conidia (Figure 1A), as well as in the cytoplasm of the hyphae and forming granular structures on the surface of hyphal stretches (Figure 1B,C).

The binding of the mAb M86 to α-Gal epitopes in *A. fumigatus* was further confirmed by flow cytometry (Figure 1D [24]). Different α-Gal levels were recorded by flow cytometry in different proportions of the conidia population [24]. The association of α-Gal to fungal proteins was assessed by an inhibition ELISA in which the reactivity of mAb M86 was measured following a pre-incubation with proteins extracted from Ascomycota species, including *A. fumigatus*, *Aspergillus nidulans*, *Candida glabrata*, *Candida albicans*, *Microsporum canis*, *Penicillium* sp., *Scedosporium* sp., and *Trichophyton benhamiae* and the Zygomycota fungi *Mucor* sp. and *Rhizopus* sp. A significant inhibition of M86 binding to Galα1-3Gal-HSA was observed after incubation with increasing concentrations of *A. fumigatus* and *Mucor* sp. proteins which suggests that among the tested fungi, α-Gal is only present in *A. fumigatus* and *Mucor* sp. (Figure 1E).

### 3.2. Oral Administration of E. coli O86:B7 Reduces Clinical Signs of Aspergillosis and Development of Lung Granulomas in A. fumigatus-Infected Turkeys

**Figure 2:**
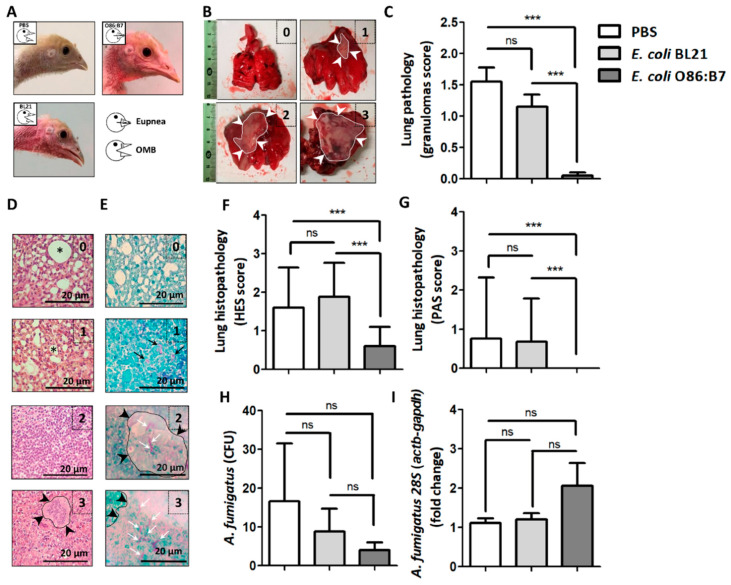
Oral administration of *E. coli* O86:B7 protects turkeys against aspergillosis. Clinical examination revealed that *A. fumigatus* infection produces open-mouthed breathing (OMB) in turkeys treated with PBS or *E. coli* BL21. Turkeys treated with *E. coli* O86:B7 were protected from developing OMB (**A**). Pulmonary lesions (i.e., granulomas, delimited area and white arrow heads) were scored (see methods). Examples of lungs with scores 0 to 3 are shown (**B**). Granuloma score was lower in turkeys treated with *E. coli* O86:B7 (**C**). Lung samples were processed for histopathology and stained with hematoxylin-eosin-saffron (HES, **D**) and periodic acid-schiff (PAS, **E**). Histological lesions were scored (see methods). Examples of histopathology samples with scores 0 to 3 are shown. Visible peribronchial regions (asterisk) and granulomas (delimited area and black arrow heads) are shown (HES score, **D**). The presence of fungal germ-tube/hyphae (black arrows) and mycelium (white arrows) was scored (see methods). Granulomas associated with fungal hyphae (delimited area and black arrow heads) are shown (PAS score, **E**). HES and PAS scores were lower in turkeys treated with *E. coli* O86:B7 (**F**,**G**). The presence of viable *Aspergillus* in lungs was quantified by colony-forming unit (CFU) counting assay (**H**). Fungal DNA levels were measured by *A. fumigatus*-specific *28S* qPCR normalizing against turkey *actb* and *gapdh* as host genes using the 2^−ΔΔ*C*t^ ratio method. Results are relative to *28S* levels in the control group (i.e., PBS) (**I**). No significant change was observed in the amount of CFU and *28S* fold change (**H**,**I**). Size of bars is indicated. 100X magnification. Results shown are means and standard deviation values. Results were compared by One-way ANOVA with Dunnett’s multiple comparison test applied for individual comparisons (*** *p* < 0.0001; ns: not significant, 2 experiments, *n* = 30).

Daily clinical examination of challenged birds revealed that oral administration of highly α-Gal expressing *E. coli* O86:B7 protects the turkeys from developing respiratory clinical signs associated with avian aspergillosis such as open-mouthed breathing (OMB, Figure 2A), gasping and hyperpnea (Supplementary Video S1). This was not the case for PBS-treated (Figure 2A and Supplementary Video S2) nor *E. coli* BL21-treated (Figure 2A and Supplementary Video S3) turkeys. Four-days post-infection, all birds were sacrificed. Assessment of macroscopic lung lesions (Figure 2B) showed lungs of turkeys treated with PBS and *E. coli* BL21 with lesions suggesting granulomas that in some cases covered the whole organ (score 3, Figure 2B). This resulted in lesional scores significantly higher than those of *E. coli* O86:B7-treated animals in which granulomas were scarce (Figure 2C). Notably, only one turkey developed a small granuloma (score 1, Figure 2B) in the right lung. The scoring of histopathological lung lesions considered the level of inflammation and granulomas assessed by HES staining (HES score, Figure 2D) and the presence of hyphae assessed by PAS (PAS score, Figure 2E). Animals treated with *E. coli* O86:B7 had significant lower inflammation (Figure 2F) and hyphae (Figure 2G) scores than those treated with PBS and *E. coli* BL21. Lung samples were homogenized and applied either on agar for CFU counting assay or used for *28S* DNA quantification by qPCR. Despite a tendency of *A. fumigatus* CFU to decrease (Figure 2H) and *28S* DNA levels to increase (Figure 2I) in the *E. coli* O86:B7-treated group, no significant differences were observed between groups. However, some animals in the *E. coli* O86:B7-treated group had 2-fold increase in the *28S* levels, possibly related to the presence of damaged fungal elements.

### 3.3. Oral Administration of E. coli O86:B7 Decreases Anti-α-Gal IgA Production in the Lungs of A. fumigatus-Infected Turkeys

Natural Abs have affinity for different α-Gal-related antigens, including Galα1-3Gal disaccharide and Galα1-3Galβ1-4GlcNAc trisaccharide. Sera levels of immunoglobulin Y (IgY) and IgA against Galα1-3Gal and Galα1-3Galβ1-4GlcNAc were measured by ELISA in sera from turkey that received PBS only. The levels of circulating IgY against Galα1-3Gal did not change over time (Supplementary Figure S1A), while the levels of anti-Galα1-3Galβ1-4GlcNAc IgY significantly increased at day 31 (Supplementary Figure S1B). Only residual levels of circulating IgA against Galα1-3Gal were detected, and the level of these Abs did not changed over time (Supplementary Figure S1C).

**Figure 3:**
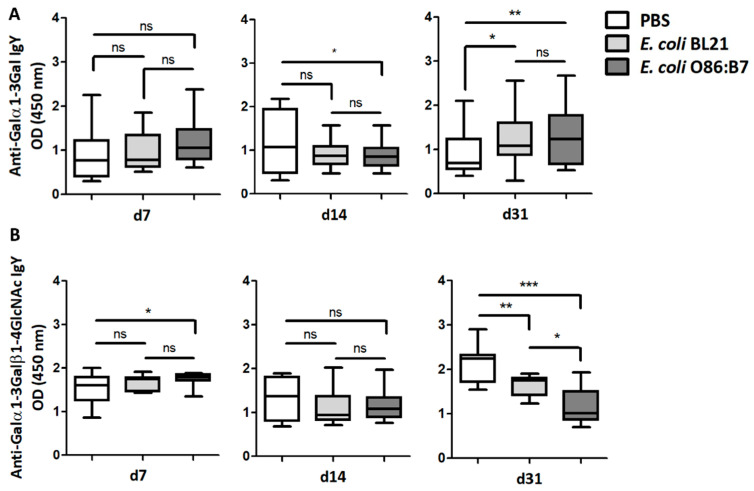
Oral administration of *E. coli* O86:B7 induces a significant decrease in the levels of anti-Galα1-3Galβ1-4GlcNAc IgY Abs in *A. fumigatus*-infected turkeys. The levels of circulating anti-α-Gal IgY Abs to Galα1-3Gal (**A**) and Galα1-3Galβ1-4GlcNAc (**B**) were measured by ELISA. Anti-Galα1-3Gal IgY Abs increased in the sera of turkeys treated with *E. coli* O86:B7 and *E. coli* BL21. Oral administration of *E. coli* O86:B7 produces a significant reduction in anti-Galα1-3Galβ1-4GlcNAc IgY Abs when compared with turkeys that were treated or not *E. coli* BL21. Results shown are means and standard deviation values. Results were compared by One-way ANOVA with Dunnett’s multiple comparison test applied for individual comparisons (* *p* < 0.05, ** *p* < 0.001, *** *p* < 0.0001; ns: not significant, 2 experiments, *n* = 30 and three technical replicates per sample).

Turkeys that received *E. coli* O86:B7 or *E. coli* BL21 orally showed no change in the levels of circulating IgY against Galα1-3Gal, and only those treated with *E. coli* O86:B7 showed higher levels of circulating IgY against Galα1-3Galβ1-4GlcNAc at day 7. The turkeys that received *E. coli* O86:B7 showed lower levels of circulating IgY against Galα1-3Gal at day 14 than turkeys that received PBS (Figure 3A), an effect not observed in turkeys that received *E. coli* BL21. At day 31, anti-Galα1-3Gal IgY levels were higher in animals that received *E. coli* O86:B7 and *E. coli* BL21, compared with animals that received PBS. However, turkeys treated with *E. coli* O86:B7 showed lower levels of anti-Galα1-3Galβ1-4GlcNAc IgY at day 31 than animals that received *E. coli* BL21 or PBS (Figure 3B).

The specificity of the reactivity of turkey sera to Galα1-3Gal was tested by enzymatic removal of terminal α-Gal residues from Galα1-3Gal-HSA. A significant decrease in the sera reactivity after the enzymatic removal of terminal α-Gal residues from Galα1-3Gal-HSA antigen was observed in turkeys from all groups (Supplementary Figure S2).

**Figure 4:**
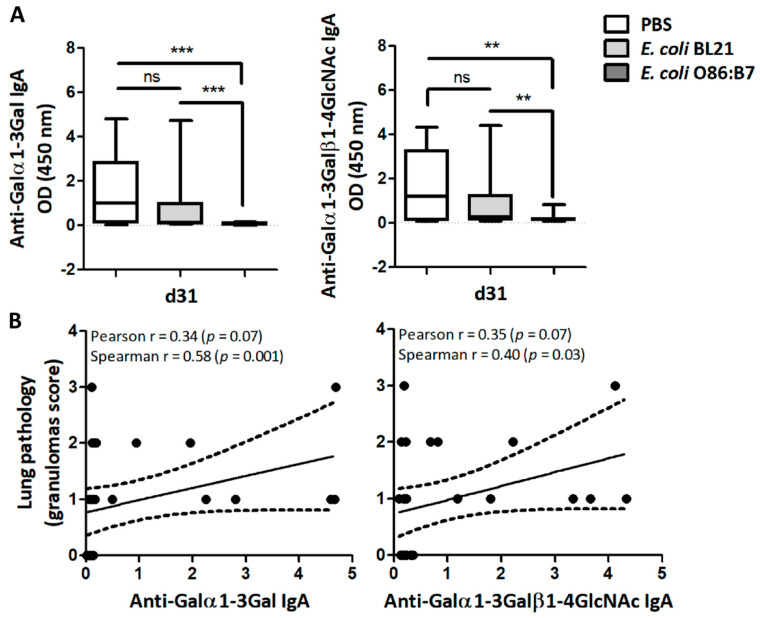
Oral administration of *E. coli* O86:B7 decreases the levels of anti-α-Gal IgA Abs which correlates with lung pathology in *A. fumigatus*-infected turkeys. The levels of IgA against α-Gal, Galα1-3Gal and Galα1-3Galβ1-4GlcNAc in lungs of *A. fumigatus*-infected turkeys were measured by ELISA. The levels of anti-α-Gal IgA between groups were compared by one-way ANOVA with Dunnett’s multiple comparison test applied for individual comparisons (** *p* < 0.001, *** *p* < 0.0001; ns: not significant, 2 experiments, *n* = 30 and three technical replicates per sample) (**A**). Correlation between the levels of anti-Galα1-3Gal and anti-Galα1-3Galβ1-4GlcNAc IgA and granuloma scores of turkeys treated with PBS, *E. coli* O86:B7 and *E. coli* BL21. Pearson and Spearman coefficients *r* and *p* values are indicated (**B**).

Lung proteins obtained from lung samples at day 31 (four days after infection) allowed the detection of IgA against Galα1-3Gal and Galα1-3Galβ1-4GlcNAc by ELISA. Only residual levels of anti-Galα1-3Gal and anti-Galα1-3Galβ1-4GlcNAc IgA Abs were detected in the lungs of turkeys that received *E. coli* O86:B7 (Figure 4A). This was not the case for *E. coli* BL21-treated or PBS-treated turkeys (Figure 4A). Positive correlations between the levels of anti-Galα1-3Gal or anti-Galα1-3Galβ1-4GlcNAc IgA and the granulomas score in the lungs were found (Figure 4B). There was no correlation between levels of anti-Galα1-3Gal and anti-Galα1-3Galβ1-4GlcNAc IgY and normalized *A. fumigatus 28S* gene levels.

### 3.4. Immunization against Galα1-3Gal Increases Fungal Development in the Lungs

**Figure 5:**
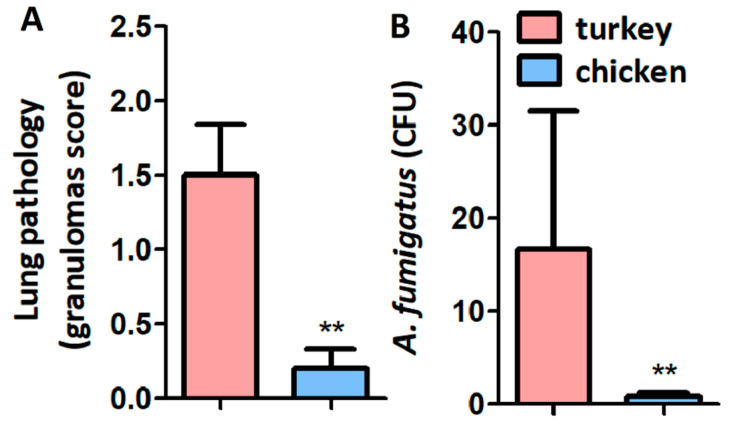
Granuloma score and CFU in turkeys and chickens infected with *A. fumigatus***.** Lung granuloma score (**A**) and CFU counting (**B**) were lower in chicken than in turkeys (**A**). Results shown are means and standard deviation values. Results were compared by Mann-Whitney U test (** *p* < 0.001; 1 experiment with chickens, *n* = 5 and 2 experiments with turkeys, *n* = 10).

Four days after infection, infected PBS-immunized turkeys developed significantly more lung granulomas (Figure 5A) and higher CFU (Figure 5B) when compared with chickens.

**Figure 6:**
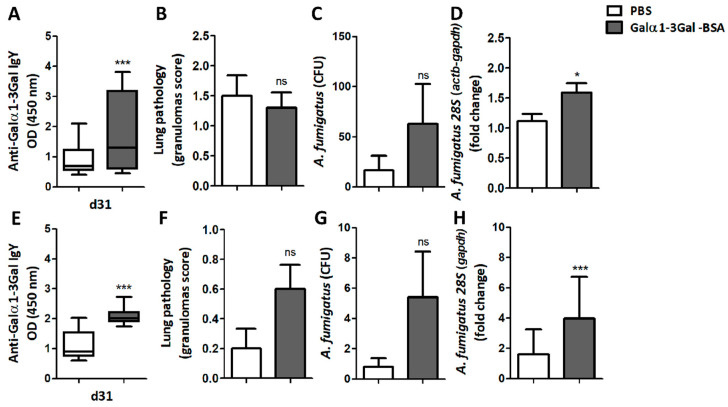
Immunization against α-Gal-BSA increases fungal burden in turkeys and chickens. The levels of circulating anti-α-Gal IgY Abs to Galα1-3Gal, lung granuloma score and *A. fumigatus* CFU number and *28S* levels in lungs, were quantified in turkeys (**A**–**D**) and chickens (**E**–**H**). Immunization against α-Gal-BSA increases the levels of anti-α-Gal IgY Abs to Galα1-3Gal and *A. fumigatus 28S* levels in turkeys (**A**,**D**) and chicken (**E**,**H**). Results shown are means and standard deviation values. Results were compared by unpaired non-parametric Mann–Whitney U test (* *p* < 0.0001, *** *p* < 0.0001; ns: not significant, 1 experiment with chicken, *n* = 10 and 2 experiments with turkeys, *n* = 20 and three technical replicates per sample in the ELISA (**A**,**B**) and qPCR (**D**,**H**) assays).

Immunization of turkeys using synthetic Galα1-3Gal conjugated to BSA (α-Gal-BSA), elicited the production of circulating IgY with affinity for Galα1-3Gal (Figure 6A and Supplementary Figure S3A). This Ab production was not associated with a significant change in the granulomas score (Figure 6B), and despite a tendency to increase, no significant difference was observed in the CFU number between the α-Gal-BSA-immunized group and the control group (Figure 6C). Furthermore, the normalized levels of *A. fumigatus 28S* were significantly higher in the lungs of turkeys immunized with α-Gal-BSA (Figure 6D), compared with the control group. No significant changes were observed in IgY production against Galα1-3Galβ1-4GlcNAc (Supplementary Figure S3B), in the levels of serum anti-Galα1-3Gal IgA (Supplementary Figure S3C), in the lung levels of anti-Galα1-3Galβ1-4GlcNAc IgA (Supplementary Figure S3D) or of anti-Galα1-3Gal IgA Abs (Supplementary Figure S3E).

In chickens, α-Gal-BSA immunization also elicited a significant increase in circulating anti-Galα1-3Gal IgY Abs (Figure 6E and Supplementary Figure S3F) and circulating anti-Galα1-3Gal IgA Abs in immunized chicken remained similar to the control group (Supplementary Figure S3G). Of the five chickens immunized with α-Gal-BSA, four developed granulomas, two in the right and left lungs and two in the left lung only. In contrast, one animal of the control group developed granulomas in the right and left lungs. Despite a tendency to increase, no significant difference was observed in the granulomas score (Figure 6F), nor in the CFU number (Figure 6G), between the α-Gal-BSA-immunized group and the control group. Notably, as per turkeys (Figure 6D), immunized chickens had a significant increase in the levels of *A. fumigatus 28S* in lungs (Figure 6H).

### 3.5. Immunization against Galα1-3Gal Is Associated with Upregulation of Pro-Inflammatory Cytokine Genes in A. fumigatus-Infected Turkeys and Chickens

We wondered whether oral administration of *E. coli* O86:B7 has an effect on the expression of genes encoding for pro-inflammatory (i.e., IFNγ, IL6, IL2) and anti-inflammatory (i.e., IL10) cytokines, as well as on the expression of innate immune receptor genes (i.e., *MyD88*) in the ceca and lungs of *A. fumigatus*-infected turkeys. *MyD88* transcription was also assessed in turkeys treated with *E. coli* BL21 or immunized with α-Gal-BSA. The effect of α-Gal-BSA immunization on the expression of pro-inflammatory cytokines (i.e., IL6 and IL2) and of MyD88 adaptor in the lungs of *A. fumigatus*-infected chicken was also tested.

**Figure 7:**
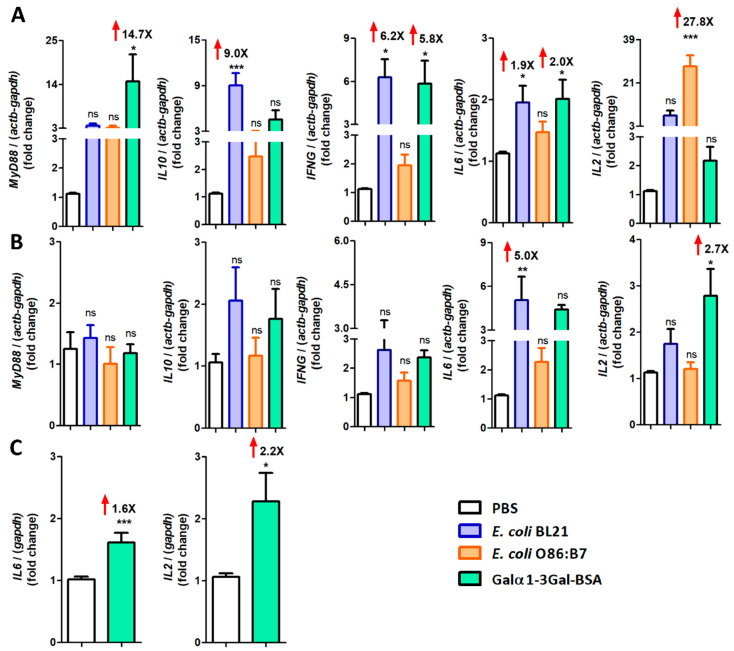
Expression of turkey and chicken cytokine genes in response to oral administration of *E. coli* O86:B7 and *E. coli* BL21 and α-Gal-BSA immunization. The figure displays the mRNA expression levels of *INFγ*, *IL6*, *IL2*, *IL10* and *MyD88* in ceca (**A**) and lungs (**B**) of turkeys and *IL6* and *IL2* in lungs of chicken (**C**). Total RNA was extracted and gene expression levels were measured by qPCR normalizing against turkey *actb* and *gapdh* as housekeeping genes, using the using the 2^−ΔΔ*C*t^ ratio method. Expression levels are relative to the control group (i.e., PBS). Results shown are means and standard deviation values. Results were compared by One-way ANOVA with Dunnett’s multiple comparison test applied for individual comparisons (* *p* < 0.0001, ** *p* < 0.001, *** *p* < 0.0001; ns: not significant, the magnitude of significant fold changes compared with the PBS are shown (red arrows), 1 experiment with chicken, *n* = 10 and 2 experiments with turkeys, *n* = 40 and three technical replicates per sample).

After cDNA normalization with the PBS control group, *MyD88*, *IFNγ* and *IL6* expression was significantly upregulated in the ceca of α-Gal-BSA-immunized turkeys (Figure 7A). α-Gal-BSA immunization also induced a significant upregulation of *IL2* in turkey (Figure 7B) and chicken lungs and *IL6* only in chicken lungs (Figure 7C). The oral administration of *E. coli* BL21 in turkeys was associated with the upregulation of *IL10*, *IFNγ* and *IL6* expression in ceca (Figure 7A) and *IL6* expression in lungs (Figure 7B). Notably, except for *IL2* for which a 27.8-fold increase was observed in ceca (Figure 7A), oral administration of *E. coli* O86:B7 was not associated with significant changes in the mRNA levels of the tested genes (Figure 7A,B).

## 4. Discussion

Evidence for the protective role of gut microbiota against pathogens expressing α-Gal on their surface was initially provided by Yilmaz et al. (2014) [8], who showed that gut colonization by *E. coli* O86:B7 expressing high levels of α-Gal elicited the production of anti-α-Gal IgM Abs that protected α-Gal-deficient mice against malaria transmission by *Anopheles* mosquitoes. In the present study, we extend these initial observations by showing that the oral administration of *E. coli* O86:B7 protects non-mammalian vertebrates against a fungal pathogen expressing α-Gal on its surface. In our model, oral administration of *E. coli* O86:B7 protected turkeys from developing clinical and lesional aspergillosis. However, in contrast with the results by Yilmaz et al. (2014) [8], the protective effect of *E. coli* O86:B7 was not associated with an increase in the levels of anti-α-Gal Abs, but with a significant reduction in the levels of circulating IgY Abs with reactivity to Galα1-3Galβ1-4GlcNAc and IgA Abs with reactivity to Galα1-3Galβ1-4GlcNAc and Galα1-3Gal in the lungs of *A. fumigatus*-infected animals. It is noteworthy that Yilmaz et al. (2014) [8] administered *E. coli* O86:B7 (~10^7^ CFU) 3 times at two weeks intervals, while we used three consecutive administrations of *E. coli* O86:B7 (~10^9^ CFU) repeated three times at four-day intervals. Our results suggest that the continuous administration of large doses of highly α-Gal expressing *E. coli* O86:B7 decreased or totally abrogated responsiveness to the α-Gal on the surface of *A. fumigatus*. Intestinal microbiota has profound effects on the gut immune system and the induction and maintenance of oral and systemic tolerance [40,41,42]. For example, oral administration of *Lactococcus lactis* engineered to secrete deamidated DQ8 gliadin epitope or ovalbumin (OVA) induced suppression of local and systemic responses to these antigens [43,44]. Likewise, intestinal colonization of mice with non-pathogenic *E. coli* expressing OVA on the surface induced the expansion of antigen-specific regulatory T cells (Tregs) and mediated systemic immune tolerance [45]. A population of Tregs with suppressive properties similar to that of mammalian Tregs was described in turkeys, chickens and ducks [46,47,48]. These avian Tregs can migrate and be resident in cecum and lung tissues [46,48]. Among the regulatory functions of Tregs is the suppression of production of antigen-specific Abs [49]. Further studies should address whether the decreased of anti-α-Gal Abs in response to *A. fumigatus* infection is caused by the induction of α-Gal-specific Tregs in *E. coli* O86:B7-treated turkeys.

Interaction between host anti-α-Gal Abs and pathogens expressing α-Gal might play a role in benefit of pathogen survival, as shown for the blood isolate #21 of *Serratia marcescens*, where binding of anti-α-Gal Abs to the bacterial lipopolysaccharide (LPS) blocked alternative complement pathway (ACP)-mediated lysis of the bacteria [50]. Likewise, depletion of inhibitory serum anti-α-Gal Abs by a soluble trisaccharide-polylysine conjugate (commercial name RA-01, www.remabtx.com) protected patients from multidrug-resistant Gram-negative bacteria, including *E. coli*, *Pseudomonas aeruginosa*, and *Klebsiella pneumonia* expressing α-Gal [50,51,52]. In the present study, *A. fumigatus* was the sole Ascomycota expressing α-Gal. *Aspergillus fumigatus* conidia is susceptible to ACP activation [53]. Therefore, the synthesis of α-Gal and binding of anti-α-Gal Abs on the surface of *A. fumigatus* may be an ultimate fungal strategy for ACP evasion in animals lacking endogenous α-Gal and capable of producing inhibitory natural anti-α-Gal Abs. The increase of *A. fumigatus* levels after α-Gal-BSA immunization in turkeys and chickens supports a role of circulating anti-Galα1-3Gal IgY Abs in promoting fungal development, by a mechanism that remains to be elucidated, but likely involving the inhibition of ACP activation.

Our results showed a positive correlation between the levels of anti-Galα1-3Galβ1-4GlcNAc and anti-Galα1-3Gal IgA and the occurrence and development of granulomas in the lungs of *A. fumigatus*-infected turkeys, suggesting a pro-inflammatory role of anti-α-Gal IgA. To further evaluate the role of anti-α-Gal Abs in aspergillosis, we took advantage of the availability of susceptible and tolerant models of avian aspergillosis. Vulnerability to this fungal infection varies among bird species, with turkeys having the highest susceptibility when compared to chickens [30,33,34]. The results confirmed that α-Gal immunization increased the number of granulomas in chickens and the fungal burden in chickens and turkeys. B cells and antibodies are required for granuloma formation in early infection by *Schistosoma japonicum* [54] and *Mycobacterium tuberculosis* [55,56,57]. Granuloma plays a role as an immune and physical barrier and is crucial in preventing pathogen dissemination within the host [58]. However, within the granulomatous lesion, the pathogen is protected from total clearance by the immune system [58,59], and, as observed in the present study, granulomas can be harmful to the host physiology by decreasing lung capacity. Respiratory impairment due to lung granulomas in aspergillosis can be lethal in turkeys [30]. Disease tolerance is the reduction of the negative impact of an infection on host fitness without directly affecting the pathogen burden [60,61]. Anti-α-Gal Abs are immune mediators of inflammation that activate the complement and macrophages, and induce NK cell recruitment and endothelial cell activation [51,62,63]. Oral administration of *E. coli* O86:B7 reduced the occurrence and severity of lung granulomas, with no effect in the fungal burden, suggesting a mechanism in which gut microbiota promotes disease tolerance in the lungs by preventing the upregulation of pro-inflammatory cytokines (i.e., IL2, IL6 and INFγ) and by decreasing the levels of anti-α-Gal Abs in response to *A. fumigatus* infection.

Previous studies showed that α-Gal immunization elicits an anti-α-Gal Abs response that protects α-Gal-deficient mice against *Trypanosoma cruzi* [64,65], *Leishmania* spp. [66], and *Plasmodium* spp. [8], and zebrafish against mycobacteria [67]. The study by Yilmaz et al., 2014 [8] showed that the protective role of anti-α-Gal Abs against malaria was not observed when *Plasmodium* sporozoites were inoculated intravenously, suggesting that the protective effect of α-gal immunization was only exerted when the pathogen was delivered in the dermis by mosquito bites. In addition, α-Gal immunization protected α-Gal-deficient mice against intraperitoneal infection with *Leishmania infantum*, subcutaneous infection with *L. amazonensis* [66] or intraperitoneal infection with *Trypanosoma cruzi* [65]. Based on this evidence, the use of α-Gal in a single-antigen pan vaccine to control major infectious diseases caused by pathogens expressing α-Gal on their surface was proposed [68,69]. Here, however, we showed that α-Gal immunization elicits an α-Gal-specific IgY response that did not protect chickens or turkeys against an intratracheal challenge with *A. fumigatus*. In addition, anti-α-Gal IgA seems to be involved in the formation and development of granulomas in birds. In contrast, α-Gal immunization reduced the mean number of tuberculous granuloma lesions in zebrafish infected with *Mycobacterium marinum* intraperitoneally [67]. However, the reduction of tuberculous granuloma was no longer observed when zebrafish were infected by mucosal *M. marinum* [67]. These studies suggest that the protective effect of α-Gal immunization varies with the route of pathogen infection.

## 5. Conclusions

In the present study, we showed that gut microbiota bacteria expressing high levels of α-Gal protect turkeys against aspergillosis. Continuous administration of *E. coli* O86:B7 abrogated the anti-α-Gal IgA response in the lungs of turkeys infected by *A. fumigatus*, a pathogen containing α-Gal on the surface. The absence of lung lesions in turkeys treated with *E. coli* O86:B7 and infected with *A. fumigatus* suggests that anti-α-Gal IgA are pro-inflammatory Abs that enhance the occurrence and development of lung lesions associated with acute aspergillosis. The mechanism by which gut microbiota abrogates anti-α-Gal IgA response in the lungs remains to be elucidated, but we hypothesized that it involves the generation of α-Gal-specific Tregs in the guts, which can then migrate to the lungs and induce tolerance to the *A. fumigatus* α-Gal. The absence of lung lesions allowed the animals to tolerate the fungal infection with no clinical signs, which suggests the possibility of using gut microbiota bacteria expressing high levels of α-Gal to prevent acute aspergillosis in animals and humans. We concluded that the increase in the level of circulating IgY against Galα1-3Gal after α-Gal-immunization, together with the presence of anti-α-Gal IgA Abs in the lungs, enhances the fungal burden, and the occurrence of granulomas in the lungs of infected chicken. The results of this study support the use of α-Gal expressing probiotic-based vaccines to modulate the α-Gal immunity [70], without the potential negative effects associated with other conventional vaccines using α-Gal as antigen. We hypothesized that a probiotic-based vaccine containing high levels of α-Gal would boost the levels of circulating anti-α-Gal IgM and/or IgG [8], which are protective against *Plasmodium* spp. [8], *T. cruzi* [65,66], *Leishmania* spp. [66], mycobacteria [68] and potentially other pathogens expressing α-Gal. At the same time, this α-Gal-probiotic-based vaccine would abrogate the anti-α-Gal IgA response in the lungs, which could then induce tolerance to diseases, such as aspergillosis and tuberculosis associated with the formation of granulomas.

## Figures and Tables

**Table 1:** Primers used in this study.

Gene	Target Species	NCBI Target Gene(s)	Forward Primer	Reverse Primer	Target Length
*gapdh*	Chicken Turkey	NM_204305 NM_001303179	CCACATGGCATCCAAGGAGT	CTCCAACAAAGGGTCCTGCT	74 bp
*β-actin*	Chicken Turkey	L08165.1 AY942620.1	GAGAAATTGTGCGTGACATCA	CCTGAACCTCTCATTGCCA	114 bp
*IL2*	Chicken	AF017645	TTGGCTGTATTTCGGTAGCA	TCCTGGGTCTCAGTTGGTGT	160 bp
	Turkey	AJ007463	GAGCATCGCTATCACCAGAA	GCAGAGTTTGCTGACTGCAC	141 bp
*IL6*	Chicken Turkey	AJ309540 XM_003207130	AGGGCCGTTCGCTATTTGAA	ACGGAACAACACTGCCATCT	112 bp
*IL10*	Turkey	NM_001303189	GCTGCGCTTCTACACAGATG	TCCCGTTCTCATCCATCTTC	203 bp
*IL4*	Turkey	NM_001303181.1	AGAGCTCATTGCCTCCACAC	ATTGCAAGGGACCTGCTCTC	72 bp
*MyD88*	Turkey	XM_019616228.1	TTACGAAGGAAGCAGCAGGAG	TGGCAAGACATCCCGATCAA	208 bp
*IFN-γ*	Turkey	XM_003202048	CTGAAGAACTGGACAGAGAG	CACCAGCTTCTGTAAGATGC	264 bp
*28S*	*A. fumigatus*	NG_055745.1	CTCGGAATGTATCACCTCTCGG	TCCTCGGTCCAGGCAGG	29 bp

## Supplementary Materials

The following are available online at https://www.mdpi.com/2076-393X/8/2/285/s1, Figure S1: Levels of circulating IgY against Galα1-3Gal (A), circulating IgY against Galα1-3Galβ1-4GlcNAc (B) and circulating IgA against Galα1-3Gal (C) were measured by indirect ELISA in control turkeys treated with PBS, Figure S2: The specificity of turkey anti-α-Gal Abs was tested by indirect ELISA in animals treated with PBS, *E. coli* BL21 and *E. coli* O86:B7. In comparison with the untreated group, a reduction in reactivity against Galα1-3Gal-HSA was observed after the antigen was pretreated with α-galactosidase, Figure S3: Levels of circulating IgY against Galα1-3Gal (A), circulating IgY against Galα1-3Galβ1-4GlcNAc (B) and circulating IgA against Galα1-3Gal (C) were measured in turkey sera by indirect ELISA in animals immunized with α-Gal-BSA (Galα1-3Gal-BSA) or the mock vaccine (PBS). Levels of IgA against Galα1-3Gal (D), and Galα1-3Galβ1-4GlcNAc (E) were measured in turkey lungs. Levels of circulating IgY (F) and circulating IgA (G) against Galα1-3Gal were measured by indirect ELISA in sera of chickens immunized with α-Gal-BSA (Galα1-3Gal-BSA) or the mock vaccine (PBS), Video S1: Video of turkey treated with *E. coli* O86:B7 and challenged with *A. fumigatus. E. coli* O86:B7 protects the turkeys from developing OMB, gasping and hyperpnea, Video S2: Video of control turkey treated with PBS and challenged with *A. fumigatus*. Turkeys in this group developed OMB, gasping and hyperpnea, Video S3: Video of turkey treated with *E. coli* BL21 and challenged with *A. fumigatus*. Turkeys in this group developed OMB, gasping and hyperpnea.
