# Supplementary material for: Gut Microbiota Abrogates Anti-α-Gal IgA Response in Lungs and Protects against Experimental Aspergillus Infection in Poultry
Source: Vaccines (Basel). 2020 Jun 7;8(2):285. doi: 10.3390/vaccines8020285 (PMC7350254; doi:10.3390/vaccines8020285)
Supplement: Supplementary file 1 [file vaccines-08-00285-s001.zip › vaccines-808979-proof-sup-1.pdf]

vaccines

MDPI

Article

Gut Microbiota Abrogates Anti-α-Gal IgA Response in Lungs and Protects against Experimental *Aspergillus* Infection in Poultry

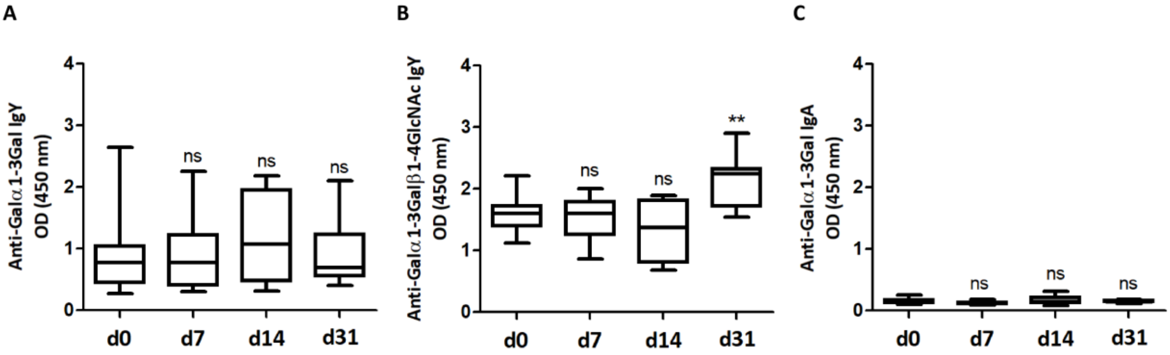

Supplementary Figure S1. Levels of circulating IgY against Galα1-3Gal (A), circulating IgY against Galα1-3Galβ1-4GlcNAc (B) and circulating IgA against Galα1-3Gal (C) were measured by indirect ELISA in control turkeys treated with PBS.

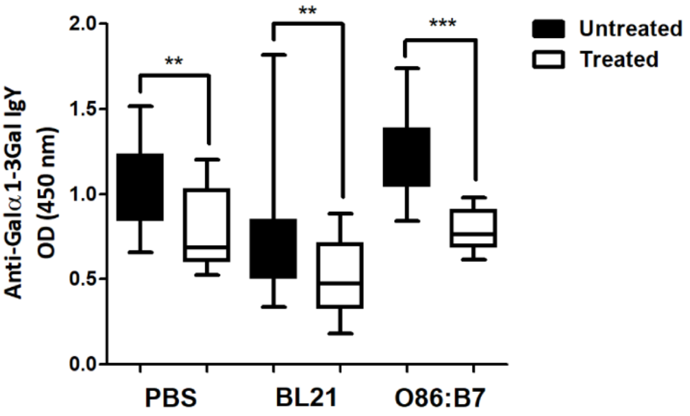

Supplementary Figure S2. The specificity of turkey anti-α-Gal Abs was tested by indirect ELISA in animals treated with PBS, E. coli BL21 and E. coli O86:B7. In comparison with the untreated group, a reduction in reactivity against Galα1-3Gal-HSA was observed after the antigen was pretreated with α-galactosidase.

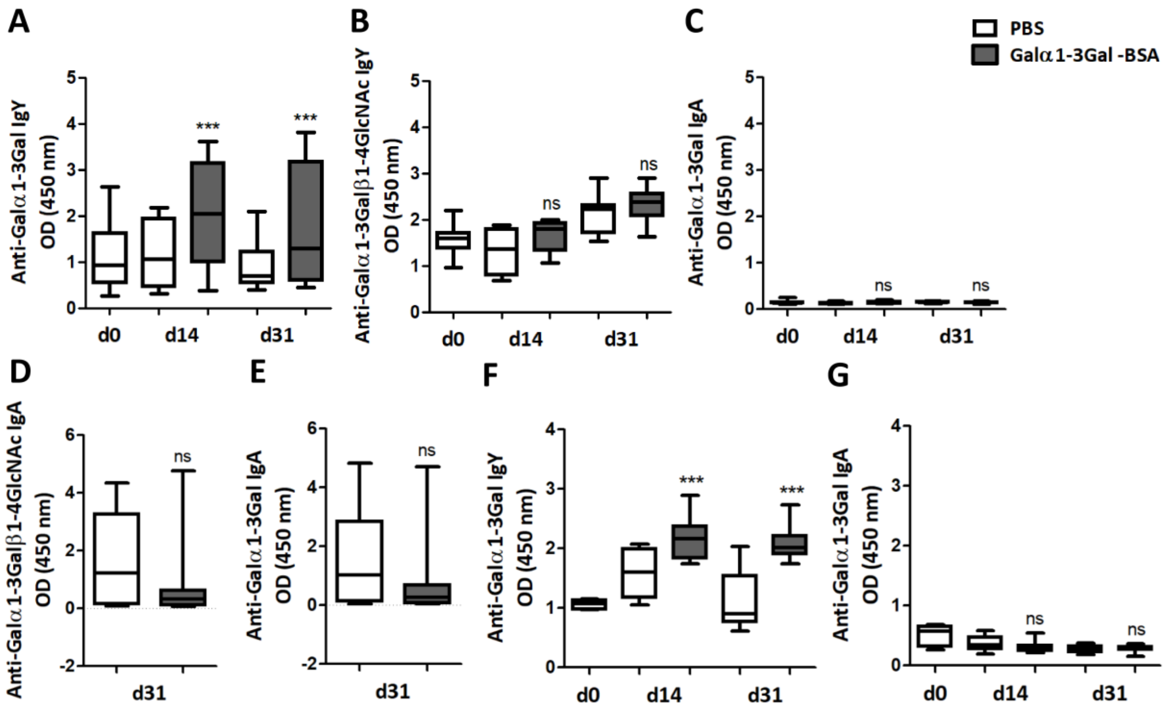

Supplementary Figure S3. Levels of circulating IgY against Galα1-3Gal (A), circulating IgY against Galα1-3Galβ1-4GlcNAc (B) and circulating IgA against Galα1-3Gal (C) were measured in turkey sera by indirect ELISA in animals immunized with α-Gal-BSA (Galα1-3Gal-BSA) or the mock vaccine (PBS). Levels of IgA against Galα1-3Gal (D), and Galα1-3Galβ1-4GlcNAc (E) were measured in turkey lungs. Levels of circulating IgY (F) and circulating IgA (G) against Galα1-3Gal were measured by indirect ELISA in sera of chickens immunized with α-Gal-BSA (Galα1-3Gal-BSA) or the mock vaccine (PBS).

© 2020 by the authors. Submitted for possible open access publication under the terms and conditions of the Creative Commons Attribution (CC BY) license (http://creativecommons.org/licenses/by/4.0/).
